# Video Assisted Thoracoscopic Treatment of Pleuropericardial Cysts

**DOI:** 10.1155/DTE.7.47

**Published:** 2001

**Authors:** F. H. W. Hermens, F. J. Visser, A. Termeer, W. B. Barendregt, J. P. Janssen

**Affiliations:** 1 Department of Pulmonary Diseases Canisius-Wilhelmina Hospital Weg door Jonkerbos 100 Nijmegen 6532 SZ The Netherlands; 2 Department of Surgery Canisius-Wilhelmina Hospital Weg door Jonkerbos 100 Nijmegen SZ 6532 The Netherlands

## Abstract

*Question of the Study* In this study, safety and feasibility of thoracoscopic fenestration of pleuropericardial cysts under local and general anaesthesia is evaluated. Besides, a rare case of a pleural cyst, causing a superior vena cava syndrome, is described.

*Materials, Patients and Methods* In a retrospective study, the results of thoracoscopic treatment of pleuropericardial cysts in three patients are presented. We performed videothoracoscopic fenestration of pleuropericardial cysts. One of these was performed under local anaesthesia. The two other cases were performed under general anaesthesia. After fenestration, talc poudrage of the inner lining of the cysts was performed in one case.

*Results* Thoracoscopic fenestration appeared to be safe and effective. No recurrence was observed. One patient was lost to follow-up.

*Answer to the Question* Thoracoscopic fenestration of pleuropericardial cysts is safe and effective. This procedure can be performed under local anaesthesia in selected cases. The role of talc poudrage of the cysts is unclear and needs further investigation.


*Diagnostic and Therapeutic Endoscopy*, Vol. 7, pp. 47–53
Reprints available directly from the publisher
Photocopying permitted by license only

© 2001 OPA (Overseas Publishers Association) N.V.
Published by license under
the Harwood Academic Publishers imprint,
part of Gordon and Breach Publishing,
a member of the Taylor & Francis Group.

### Video Assisted Thoracoscopic Treatment of Pleuropericardial Cysts

F.H.W. HERMENS[a], F.J. VISSER[a], A. TERMEER[a], W.B. BARENDREGT[b] and J.P. JANSSEN[a,*]

[a]*Department of Pulmonary Diseases;* [b]*Department of Surgery, Canisius-Wilhelmina Hospital, Weg door Jonkerbos 100, 6532 SZ Nijmegen, The Netherlands*

*(Received 23 June 2000; Revised 7 August 2000; In final form 18 September 2000)*

***Question of the Study*** **In this study, safety and feasibility of thoracoscopic fenestration of pleuropericardial cysts under local and general anaesthesia is evaluated. Besides, a rare case of a pleural cyst, causing a superior vena cava syndrome, is described.**

***Materials, Patients and Methods*** **In a retrospective study, the results of thoracoscopic treatment of pleuropericardial cysts in three patients are presented. We performed videothoracoscopic fenestration of pleuropericardial cysts. One of these was performed under local anaesthesia. The two other cases were performed under general anaesthesia. After fenestration, talc poudrage of the inner lining of the cysts was performed in one case.**

***Results*** **Thoracoscopic fenestration appeared to be safe and effective. No recurrence was observed. One patient was lost to follow-up.**

***Answer to the Question*** **Thoracoscopic fenestration of pleuropericardial cysts is safe and effective. This procedure can be performed under local anaesthesia in selected cases. The role of talc poudrage of the cysts is unclear and needs further investigation.**

*Keywords*: Interventional thoracoscopy, Pleuropericardial cyst

### INTRODUCTION

Video-assisted interventional thoracoscopy has replaced thoracotomy as the procedure of choice for diagnosis and treatment of many pleural and pulmonary diseases [1–3]. Benign mediastinal masses such as cysts often represent ideal lesions for resection using minimally invasive techniques.

Most cysts are asymptomatic and can be left untreated, as these cysts do not tend to malignant degeneration. However, sometimes, especially in children, these cysts can give complaints of chest pain or dyspnea. In these cases treatment is

* Corresponding author. Tel.: 31-24-3658755. Fax: 31-24-3658978. E-mail: jpj@knmg.nl.

indicated. Several forms of treatment have been described in the literature: complete resection by means of thoracotomy [4], videoassisted thoracoscopic surgery [2] and percutaneous aspiration under ultrasound guidance, sometimes followed by ethanol sclerosis [5].

We present three cases of pleuropericardial cysts. One pericardial cyst was localized in the right cardiophrenic angle and the other in the left cardiophrenic angle. The third patient had a pleural cyst which was localized in the right paratracheal position, which presented itself clinically as a superior vena cava syndrome. All cases were treated by means of thoracoscopic fenestration.

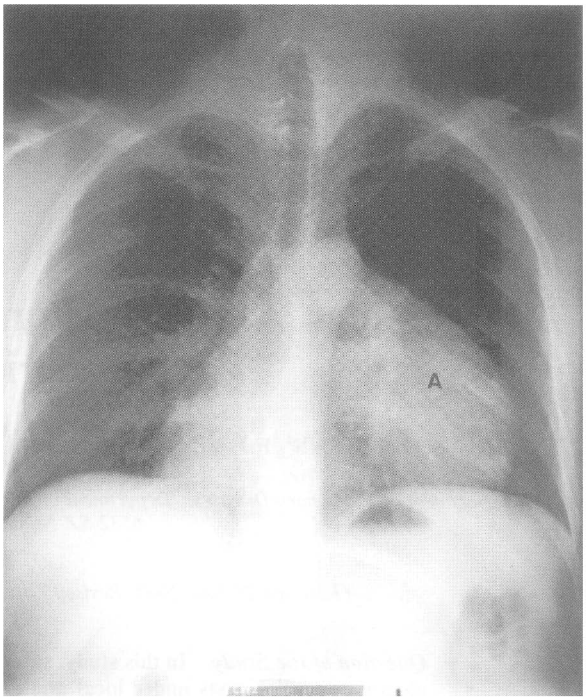

FIGURE 1 Chest roentgenogram of Case 1. A mass can be seen adjacent to the left side of the heart (A), which appeared to be a pericardial cyst after thoracoscopic treatment.

### PRESENTATION OF CASES

### Case 1

A 35 year old man presented with chest pain, which had existed for several years. The chest roentgenogram showed a mediastinal mass at the left paracardial line (Fig. 1). CT scan showed a

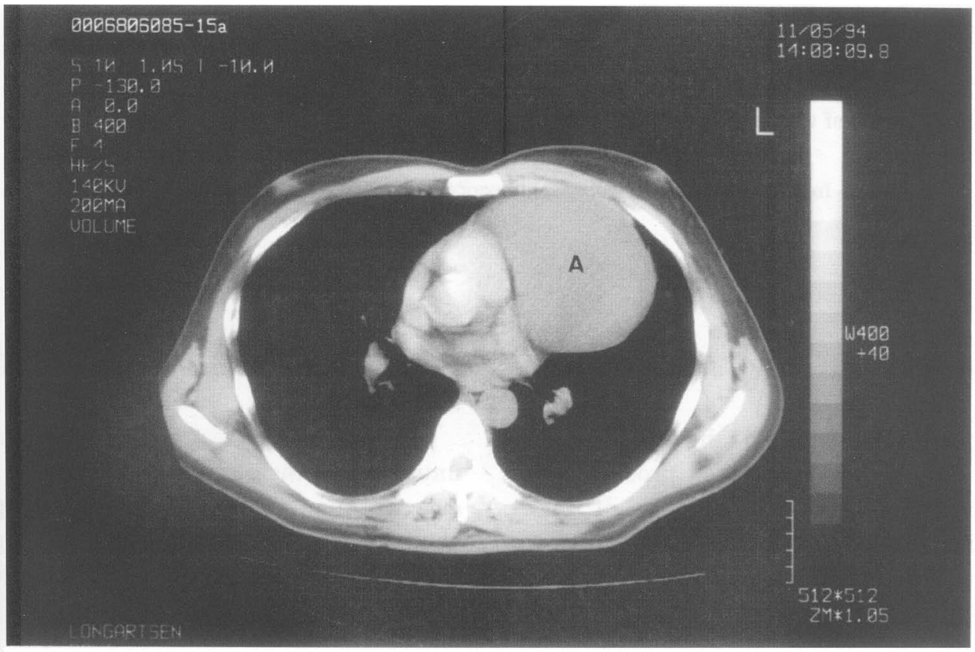

FIGURE 2 CT image of Case 1. The cyst (A) is localised at the left side of the mediastinum, adjacent to the anterior chest wall.

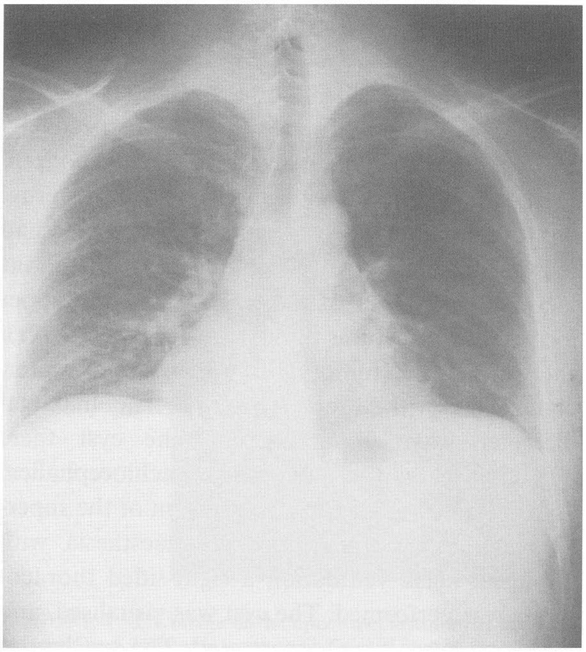

FIGURE 3 Chest roentgenogram of Case 1, 3 months after treatment.

density adjacent to the left upper mediastinum, with a homogeneous content and the density of water. The radiological diagnosis was a cyst, which had a maximum diameter of 9.5 cm (Fig. 2). The cyst extended from the diaphragm to the aortic arch. Thoracoscopy was performed under local anaesthesia. After puncture, a clear fluid escaped from the cyst, which was fenestrated afterwards. Histology confirmed the diagnosis of a pericardial cyst. The procedure was well tolerated. No complications were observed. The patients' complaints were relieved and the chest X-ray returned to normal. There were no signs of recurrence after a few months (Fig. 3). However, the patient who was a refugee, left the country thereafter and was lost to follow-up.

### Case 2

A 43 year old woman consulted our practice for an abnormal chest X-ray, which was made after an accident. A translucent density was seen in the right cardiophrenic angle. CT scan showed a cystic formation on the right side of the heart, with a diameter of 9.8 cm, diagnosed as a pericardial

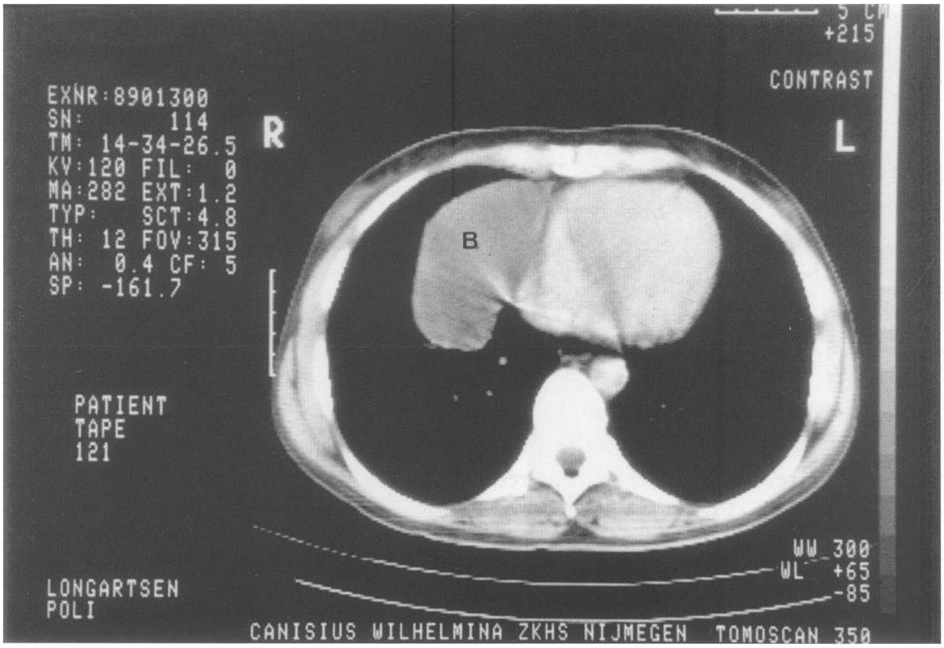

FIGURE 4 CT image of Case 2. Cystic formation adjacent to the right side of the heart (B) with the density of water.

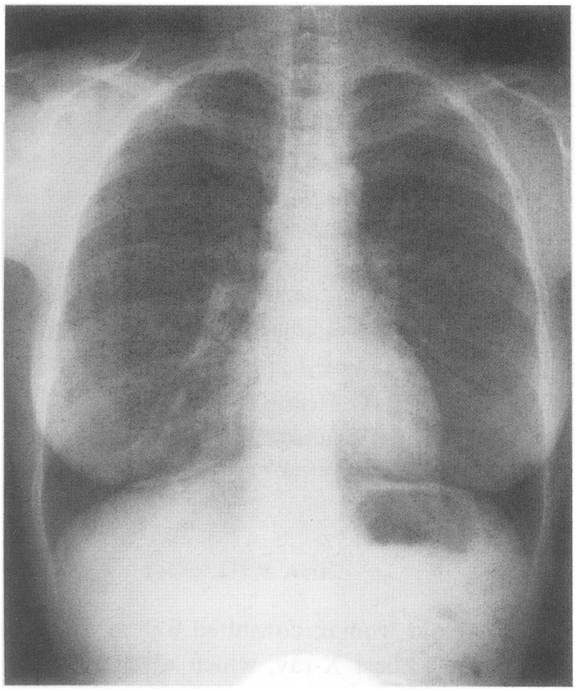

FIGURE 5 Chest roentgenogram of Case 2, 4 years after treatment.

cyst (Fig. 4). She refused a thoracotomy because of its invasive character. With the introduction of minimal invasive techniques she accepted to undergo treatment 6 years later. Under general anaesthesia with selective intubation a right sided thoracoscopy was performed. The cyst was immediately identified. After puncture a colourless fluid could be aspirated. After aspiration the cyst could be easily grasped with a forceps. With a pair of endoscopic scissors thoracoscopic fenestration was performed. Talc poudrage was accomplished inside the cyst. After fenestration the patient did not experience any complaints, nor did any complications occur. She was dismissed from hospital 3 days after the procedure. No recurrence occurred after a follow-up of 4 years (Fig. 5).

### Case 3

A 71 year old man was presented to us with paroxysmal collapse and the clinical signs of a superior vena cava syndrome. The chest roentgenogram showed a large mass on the right side of the upper mediastinum with shift of the trachea to the left side (Fig. 6). Retrospectively, this mass was visible on a chest roentgenogram 6 years before, but was mistaken for a retrosternal goitre at that time. In 6 years, the diameter had increased from 4.5 to 8.5 cm. Besides, there was increased deviation of the trachea and the upper mediastinal structures to the left side. The CT scan showed a large tumor with a density of water, compatible with a cyst (Fig. 7). The maximal diameter was 10 cm. Due to the cyst there was compression of the right brachiocephalical vein, without significant compression of the superior vena cava. Under general anaesthesia with laryngeal mask ventilation a right-sided thoracoscopy was performed. The cyst was visualised, and after puncture, it was fenestrated. The swelling of the neck veins disappeared immediately after the

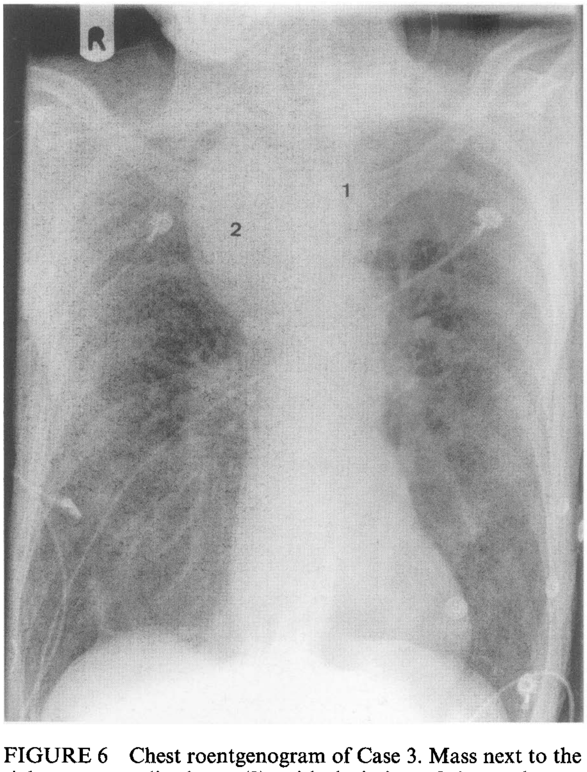

FIGURE 6 Chest roentgenogram of Case 3. Mass next to the right upper mediastinum (2), with deviation of the trachea to the left (1).

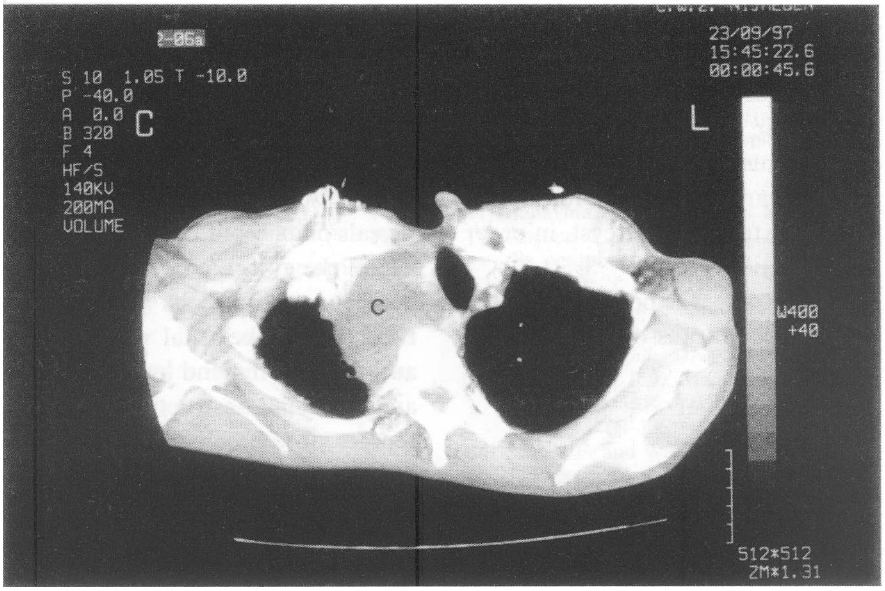

FIGURE 7 CT image of the upper mediastinum at the level of the sternoclavicular joints in Case 3. A cyst at the right side of the mediastinum (C) compresses the afferent veins of the vena cava superior. The upper mediastinum is deviated to the left.

thoracoscopy. Histologic examination confirmed the diagnosis of a pleural cyst. After a follow up of 22 months there were no signs of recurrence (Fig. 8).

### TECHNIQUE

The patient treated under local anaesthesia was premedicated with atropine 0.5 mg and morphine 10 mg. The patient was positioned in the lateral decubitus position. Local anaesthesia was obtained by local subcutaneous and intercostal infiltration of 10 mg of lidocain.

In the other two patients, thoracoscopy was performed under general anaesthesia with a double lumen tube (single lung ventilation) in the second patient and a laryngeal mask in the third patient. Although deflation of the lung is not complete in this case, it did not interfere with the procedure. A 7 mm trocar is inserted in the 5th or 6th intercostal space in the midaxillary line. After deflation of the lung, an optical telescope is introduced to inspect the lung and to visualise the cyst. Another trocar is

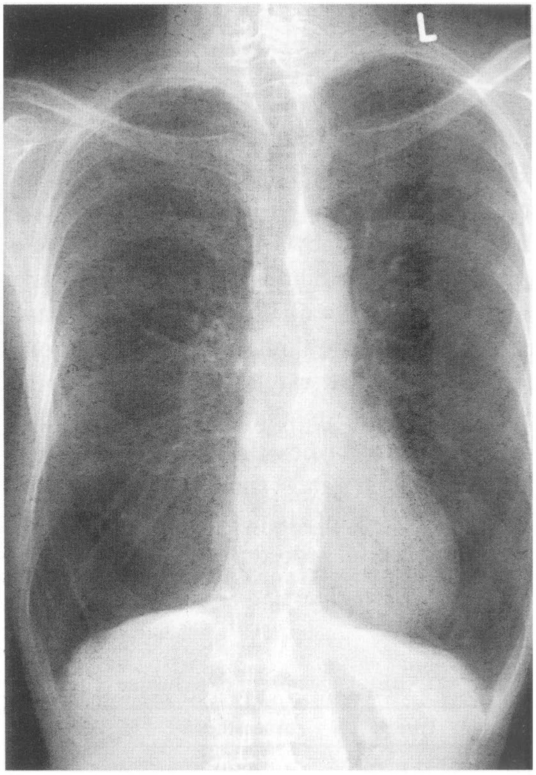

FIGURE 8 Chest roentgenogram of Case 3, 22 months after treatment.

introduced in the anterior or posterior axillary line, in the 7th or 8th intercostal space. This port is used to introduce a needle to puncture the cyst, and subsequently a pair of scissors to fenestrate the cyst. In the patients treated under general anaesthesia, a third trocar is introduced for a grasper. The grasper is used to fixate the cyst, in order to facilitate the fenestration.

In one case, talc poudrage was performed in the interior wall of the cyst by means of insufflation.

### DISCUSSION

Video-assisted thoracoscopic fenestration of a cyst is an excellent way to achieve minimally invasive surgery if surgical diagnosis or treatment is necessary. In the case of mediastinal cysts, a medline search (1966–2000) shows several recommendations, varying from doing nothing in asymptomatic patients [5], percutaneous aspiration with or without ethanol, minocyclin or doxycyclin [5,6] injection, or surgical treatment by means of interventional thoracoscopy [3,7–9] or thoracotomy [4,9–12].

Because of its minimal invasive character, interventional thoracoscopy is the preferred procedure if feasible. It has shown to be safe, effective and has expanding possibilities, even in elderly patients or children [2,3,13–15]. Especially in the case of mediastinal cysts, we think interventional thoracoscopy is an excellent way to replace thoracotomy. One author suggests that thoracoscopy should only be performed when the mass on CT image is well-encapsulated and small-to-moderate sized (<6 cm) because of better manageability [16], although successful procedures on larger ones have been reported [2,17].

We performed videothoracoscopic fenestration of cysts in three patients without specific thoracoscopic complications (Table I). The question whether or not to perform talc poudrage of the cyst if total removal is impossible remains controversial. A literature review (Medline 1996–2000) reveals no clues in this matter. We performed talcage of the cyst in one case with satisfying results.

Adherence of cysts to vital mediastinal structures can make total excision hazardous. Some authors recommend partial excision of the cyst and epithelial destruction [18,19]. There are some reports on ethanol or doxycycline with lidocaine injection with good results, but these are after percutaneous aspiration of the cyst and not after partial resection by means of thoracoscopic fenestration [5,20]. There is one report by Barman *et al.*, which mentions partial excision and ablation of the lining of the cyst by electrocautery [18], but a randomised study is not available. To our knowledge, there are no randomised studies in the literature to prove the superiority of complete excision over partial excision or puncture with chemical obliteration in terms of safety or recurrence rate (Medline search 1996–2000, search terms: mediastinal-cyst-excision).

Pleuropericardial cysts usually are congenital anomalies. After the third week of gestation, the mesoderm separates itself to form pleural, pericardial and peritoneal spaces [21]. Incomplete partitioning can result in a pleuropericardial cyst. They usually manifest themselves as an asymptomatic disease in the case of well-limited lesions, in contrast to bronchogenic cysts which often become symptomatic or complicated [2,22]. Of the pericardial cysts, 70–80% is located in the right cardiophrenic angle of which the incidence is estimated to be 1/100,000 [23,24]. Pleuropericardial cysts

TABLE I Summary of technique of treatment and results in three patients with pleuropericardial cysts

| Case | Patient | Anaesthesia | Ventilation | Talcage | Complication | Cyst | Recurrence |
|---|---|---|---|---|---|---|---|
| 1 | 33 yr male | local | spontaneous | no | no | pericardial | no follow-up |
| 2 | 33 yr female | total | double lumen | yes | no | pericardial | no (4 years) |
| 3 | 71 yr male | total | laryngeal mask | no | no | pleural | no (2 years) |

constitute 7% of all mediastinal tumors [24,25]. In our first patient, the cyst was localized in the left cardiophrenic angle. A pleural cyst like the one found in our third patient is extremely rare. A pleural cyst with obstruction of the brachiocephalical vein has not been described before (Medline search 1966–2000). In this case the diagnosis was missed on the initial chest roentgenogram, and suspected to be a cyst only after CT scanning of the thorax 6 years later. Thoracoscopy both confirmed the diagnosis and solved the problem of venous obstruction.

In conclusion, if treatment of a pleuropericardial cyst is mandatory, thoracoscopic intervention is a safe and effective method for both diagnosis and treatment. The role of talc poudrage of the inner lining of the cyst is unclear.

### *References*

[1] Bousamra, M., Haasler, G.B., Patterson, G.A. and Roper, C.L. A comparative study of thoracoscopic vs. open removal of benign neurogenic mediastinal tumors. *Chest* 1996; **109**(6): 1461–1465.

[2] Mouroux, J., Padovani, B., Maalouf, J., Bourgeon, A. and Richelme, H. Pleuropericardial cysts: Treatment by videothoracoscopy. *Surg. Laparosc. Endosc.* 1996; **6**(5): 403–404.

[3] Jaklitsch, M.T., DeCamp, M.M., Liptay, M.J. *et al.* Video-assisted thoracic surgery in the elderly. *Chest* 1996; **110**(3): 751–758.

[4] Abad, C., Rey, A., Feijoo, J., Gonzalez, G. and Martin-Suarez, J. Pericardial cyst. Surgical resection in two symptomatic cases. *J. Cardiovasc. Surg.* 1996; **37**(2): 199–202.

[5] Kinoshita, Y., Shimada, T., Murakami, Y. *et al.* Ethanol sclerosis can be a safe and useful treatment for pericardial cyst. *Clin. Cardiol.* 1996; **19**(10): 833–835.

[6] 11th National ACCP Board Review. ISBN # 0-91 6609-09-X.

[7] Kern, J.A., Daniel, T.M., Tribble, C.G., Silen, M.L. and Rodgers, B.M. Thoracoscopic diagnosis and treatment of mediastinal masses. *Ann. Thorac. Surg.* 1993; **56**: 92–96.

[8] Horita, K., Sakao, Y. and Itoh, T. Excision of a recurrent pericardial cyst using video-assisted thoracic surgery. *Chest* 1998; **114**: 1203–1204.

[9] Cioffi, U., Bonavina, L., De Simone, M. *et al.* Presentation and surgical management of bronchogenic and esophageal duplication cysts in adults. *Chest* 1998; **113**: 1492–1496.

[10] Aktogu, S., Yuncu, G., Halilcolar, H., Ermete, S. and Buduneli, T. Bronchogenic cysts: clinicopathological presentation and treatment. *Eur. respir. J.* 1996; **9**: 2017–2021.

[11] Bonavina, L., Pavanello, M., Baisi, A., Castoro, C., Ancona, E. and Peracchia, A. Mediastinal cyst involving the oesophagus: diagnosis and results of surgical treatment. *Eur. J. Surg.* 1996; **162**(9): 703–707.

[12] Feng, G., Quan, L., Shumei, X., Hua, R., Zhiyong, Z. and Zejian, L. Diagnosis and surgical treatment of bronchogenic cysts. *Chin. Med. Sci. J.* 1995; **10**(1): 61.

[13] Landreneau, R.J., Mack, M.J., Dowling, R.D. *et al.* The role of thoracoscopy in lung cancer management. *Chest* 1998; **113**: 6S–12S.

[14] Schier, F. and Waldschmidt, J. Thoracoscopy in children. *J. Pediatr. Surg.* 1996; **31**(12): 1640–1643.

[15] Schwarz, C.D., Puschmann, R., Eckmayr, J., Harti, P., Mayer, K.H. and Zisch, R.I. Videoendoscopic procedures in thoracic surgery: technical aspects and report of removal of a mediastinal cyst. *Surg. Laparosc. Endosc.* 1995; **5**(2): 94–99.

[16] Kaiser, L.R. Thoracoscopic resection of mediastinal tumors and the thymus. *Chest surg. clin. N. Am.* 1996; **6**(1): 41–52.

[17] Hazelrigg, S.R., Landreneau, R.J., Mack, M.J. and Acuif, T.E. Thoracoscopic resection of mediastinal cysts. *Ann. Thorac. Surg.* 1993; **56**: 659–660.

[18] Barman, A.A., Moideen, A.S., Chaudhry, S.S. and Reich, D. Laceration of the left pulmonary artery during removal of a bronchogenic cyst by right thoracotomy. *Chest* 1991; **100**(1): 267–268.

[19] Gourin, A., Garzon, A.A., Rosen, Y. and Lyons, H.A. Bronchogenic cysts. Broad spectrum of presentation. *NY State J. Med.* 1976; **76**(5): 714–719.

[20] Urschel, J.D. and Horan, T.A. Mediastinoscopic treatment of mediastinal cysts. *Ann. Thorac. Surg.* 1994; **58**(6): 1698–1700.

[21] Murray, J.F. and Nadel, J.A. Textbook of respiratory medicine. W.B. Saunders Company, 1994: 2146–2147.

[22] St. Georges, R., Deslauriers, J., Duranceau, A. *et al.* Clinical spectrum of bronchogenic cysts of the mediastinum and lung in the adult. *Ann. Thorac. Surg.* 1991; **52**: 6–13.

[23] Boisserie-Lacroix, M., Martigne, Ch., Laurent, F., Drouillard, J. and Grelet, Ph. A pleuropericardial cyst in an unusual location: the value of magnetic resonance. *Comp. Med. Im. Graph* 1988; **12**(5): 277–280.

[24] Le Roux, B.T. Pericardial coelemic cysts. *Thorax* 1959; **14**: 27–34.

[25] Nelson, T.C., Shefts, L.M. and Bowers, W.F. Mediastinal tumors: an analysis of 141 cases. *Dis. Chest* 1957; **32**: 123–153.

